# Supplementary material for: Up on the roof and down in the dirt: Differences in substrate properties (SOM, potassium, phosphorus and pH) and their relationships to each other between sedum and wildflower green roofs
Source: PLoS One. 2019 Dec 13;14(12):e0225652. doi: 10.1371/journal.pone.0225652 (PMC6910666; doi:10.1371/journal.pone.0225652)
Supplement: S1 Table — (DOCX) [file pone.0225652.s001.docx]

**Supporting Information 1**

(from Gabrych *et al.,* 2016)

Number of sample plots (*n*) based on green roof size. Listed are: range in green roof size, total number of plots (randomized plots located all over the roof including the central plots), and number of central plots (plot located in the middle of the roof, based on roof dimensions).

| Roof size m^2^ | *n*(plots) | *n*(central plots) |
| --- | --- | --- |
| 8-19 | 4 | 1 |
| 20-29 | 5 | 1 |
| 30-39 | 6 | 1 |
| 40-49 | 7 | 1 |
| ... | ... | ... |
| 90-99 | 12 | 1 |
| 100-199 | 13 | 1 |
| 200-299 | 14 | 1 |
| 300-399 | 15 | 2 |
| ... | ... | .... |
| 900-999 | 21 | 2 |
| 1000-1999 | 22 | 2 |
| 2000-2999 | 23 | 2 |
