# Supplementary material for: Up on the roof and down in the dirt: Differences in substrate properties (SOM, potassium, phosphorus and pH) and their relationships to each other between sedum and wildflower green roofs
Source: PLoS One. 2019 Dec 13;14(12):e0225652. doi: 10.1371/journal.pone.0225652 (PMC6910666; doi:10.1371/journal.pone.0225652)
Supplement: S2 Table — (DOCX) [file pone.0225652.s002.docx]

Supporting Information 2

**Species lists for individual roofs**

University of Brighton, Varley Hub Foyer (Sedum)

| Common name | Scientific name | Family |
| --- | --- | --- |
| Ash | *Fraxinus excelsior* | Oleaceae |
| Autumn hawk bit | *Scorzoneroides autumnalis* | Asteraceae |
| Bristly ox-tongue | *Helminthotheca echioides* | Asteraceae |
| Cat’s ear | *Hypochaeris radicata* | Asteraceae |
| Caucasian stonecrop | *Sedum spurium* | Crassulaceae |
| Common ragwort | *Senecio jacobaea* | Asteraceae |
| Common vetch | *Vicia sativa* | Fabaceae |
| Crane’s bill | *Geranium sp.* | Geraniaceae |
| Dandelion | *Taraxacum officinale* | Asteraceae |
| English stonecrop | *Sedum anglicum* | Crassulaceae |
| Groundsel | *Senecio vulgaris* | Asteraceae |
| Hop trefoil | *Trifolium campestre* | Fabaceae |
| Reflexed stonecrop | *Sedum rupestre* | Crassulaceae |
| Rosebay willowherb | *Chamerion angustifolium* | Onagraceae |
| Russian stonecrop | *Sedum kamtschaticum* | Crassulaceae |
| Smooth sow thistle | *Sonchus oleraceus* | Asteraceae |
| White clover | *Trifolium repens* | Fabaceae |
| White stonecrop | *Sedum album* | Crassulaceae |

University of Brighton, Huxley Building (Sedum)

| Common name | Scientific name | Family |
| --- | --- | --- |
| Ash | *Fraxinus excelsior* | Oleaceae |
| Birch | *Betula pendula* | Betulaceae |
| Bristly ox-tongue | *Helminthotheca echioides* | Asteraceae |
| Canadian fleabane | *Erigeron canadensis* | Asteraceae |
| Cat’s ear | *Hypochaeris radicata* | Asteraceae |
| Caucasian stonecrop | *Sedum spurium* | Crassulaceae |
| Corn marigold | *Glebionis segetum* | Asteraceae |
| Cornflower | *Centaurea cyanus* | Asteraceae |
| Crane’s bill | *Geranium sp.* | Geraniaceae |
| English stonecrop | *Sedum anglicum* | Crassulaceae |
| Groundsel | *Senecio vulgaris* | Asteraceae |
| Hemp agrimony | *Eupatorium cannabinum* | Asteraceae |
| Hop trefoil | *Trifolium campestre* | Fabaceae |
| Mexican fleabane | *Erigeron karvinskianus* | Asteraceae |
| Narrow leaved ragwort | *Senecio inaequidens* | Asteraceae |
| Prickly sow thistle | *Sonchus asper* | Asteraceae |
| Reed sp. | *Phragmites sp.* | Poeaceae |
| Reflexed stonecrop | *Sedum rupestre* | Crassulaceae |
| Ribbed plantain | *Plantago lanceolata* | Plantaginaceae |
| Rosebay willowherb | *Chamerion angustifolium* | Onagraceae |
| Russian stonecrop | *Sedum kamtschaticum* | Crassulaceae |
| Self-heal | *Prunella vulgaris* | Lamiaceae |
| Smooth sow thistle | *Sonchus oleraceus* | Asteraceae |
| Speedwell sp. | *Veronica sp.* | Plantaginaceae |
| White clover | *Trifolium repens* | Fabaceae |
| White stonecrop | *Sedum album* | Crassulaceae |
| Wild mint | *Mentha sp.* | Lamiaceae |
| Willow sp. | *Salix sp.* | Salicaceae |
| Yarrow | *Achillea millefolium* | Asteraceae |

University of Brighton, Falmer Sport’s Hall 1 (Sedum)

| Common name | Scientific name | Family |
| --- | --- | --- |
| Caucasian stonecrop | *Sedum spurium* | Crassulaceae |
| Common vetch | *Vicia sativa* | Fabaceae |
| Crane’s bill | *Geranium sp.* | Geraniaceae |
| Dandelion | *Taraxacum officinale* | Asteraceae |
| English stonecrop | *Sedum anglicum* | Crassulaceae |
| Groundsel | *Senecio vulgaris* | Asteraceae |
| Many seeded goosefoot | *Lipandra polysperma* | Amaranthaceae |
| Prickly sow thistle | *Sonchus asper* | Asteraceae |
| Purple clover | *Trifolium pratense* | Fabaceae |
| Reflexed stonecrop | *Sedum rupestre* | Crassulaceae |
| Rosebay willowherb | *Chamerion angustifolium* | Onagraceae |
| Russian stonecrop | *Sedum kamtschaticum* | Crassulaceae |
| Smooth sow thistle | *Sonchus oleraceus* | Asteraceae |
| White stonecrop | *Sedum album* | Crassulaceae |

University of Brighton, Falmer Sport’s Hall 2 (Sedum)

| Common name | Scientific name | Family |
| --- | --- | --- |
| Black medick | *Medicago lupulina* | Fabaceae |
| Bristly ox-tongue | *Helminthotheca echioides* | Asteraceae |
| Common mouse ear | *Cerastium fontanum* | Caryophyllaceae |
| Common vetch | *Vicia sativa* | Fabaceae |
| Crane’s bill | *Geranium sp.* | Geraniaceae |
| Dandelion | *Taraxacum officinale* | Asteraceae |
| English stonecrop | *Sedum anglicum* | Crassulaceae |
| Groundsel | *Senecio vulgaris* | Asteraceae |
| Many seeded goosefoot | *Lipandra polysperma* | Amaranthaceae |
| Reflexed stonecrop | *Sedum rupestre* | Crassulaceae |
| Russian stonecrop | *Sedum kamtschaticum* | Crassulaceae |
| White stonecrop | *Sedum album* | Crassulaceae |

University of Brighton, Checkland Building 4^th^ floor, north (Wildflower)

| Common name | Scientific name | Family |
| --- | --- | --- |
| Black medick | *Medicago lupulina* | Fabaceae |
| Bladder campion | *Silene vulgaris* | Caryophyllaceae |
| Bristly ox-tongue | *Helminthotheca echioides* | Asteraceae |
| Cat’s ear | *Hypochaeris radicata* | Asteraceae |
| Common mouse ear | *Cerastium fontanum* | Caryophyllaceae |
| Common vetch | *Vicia sativa* | Fabaceae |
| Creeping buttercup | *Ranunculus repens* | Ranunculaceae |
| Dandelion | *Taraxacum officinale* | Asteraceae |
| Devil’s bit scabious | *Succisa pratensis* | Caprifoliaceae |
| Fool’s parsley | *Aethusa cynapium* | Apiaceae |
| Hop trefoil | *Trifolium campestre* | Fabaceae |
| Lady’s bedstraw | *Galium verum* | Rubiaceae |
| Ox-eye daisy | *Leucanthemum vulgare* | Asteraceae |
| Perforate St. John’s wort | *Hypericum perforatum* | Hypericaceae |
| Ribbed plantain | *Plantago lanceolata* | Plantaginaceae |
| Salad burnet | *Sanguisorba minor* | Rosaceae |
| Self-heal | *Prunella vulgaris* | Lamiaceae |
| Smooth sow thistle | *Sonchus oleraceus* | Asteraceae |
| Viper’s bugloss | *Echium vulgare* | Boraginaceae |
| White clover | *Trifolium repens* | Fabaceae |
| Wild basil | *Clinopodium vulgare* | Lamiaceae |

University of Brighton, Checkland Building, 3^rd^ floor, north (Wildflower)

| Common name | Scientific name | Family |
| --- | --- | --- |
| Black medick | *Medicago lupulina* | Fabaceae |
| Bristly ox-tongue | *Helminthotheca echioides* | Asteraceae |
| Broad leaved dock | *Rumex obtusifolius* | Polygonaceae |
| Cat’s ear | *Hypochaeris radicata* | Asteraceae |
| Common corn cockle | *Agrostemma githago* | Caryophyllaceae |
| Creeping buttercup | *Ranunculus repens* | Ranunculaceae |
| Dandelion | *Taraxacum officinale* | Asteraceae |
| Groundsel | *Senecio vulgaris* | Asteraceae |
| Hop trefoil | *Trifolium campestre* | Fabaceae |
| Horseshoe vetch | *Hippocrepis comosa* | Fabaceae |
| Lady’s bedstraw | *Galium verum* | Rubiaceae |
| Lesser trefoil | *Trifolium dubium* | Fabaceae |
| Many seeded goosefoot | *Lipandra polysperma* | Amaranthaceae |
| Ox-eye daisy | *Leucanthemum vulgare* | Asteraceae |
| Petty spurge | *Euphorbia peplus* | Euphorbiaceae |
| Salad burnet | *Sanguisorba minor* | Rosaceae |
| Self-heal | *Prunella vulgaris* | Lamiaceae |
| Viper’s bugloss | *Echium vulgare* | Boraginaceae |
| White clover | *Trifolium repens* | Fabaceae |
| Wild basil | *Clinopodium vulgare* | Lamiaceae |
| Wild thyme | *Thymus serpyllum* | Lamiaceae |

University of Brighton, Checkland Building, 2^nd^ floor, north (Wildflower)

| Common name | Scientific name | Family |
| --- | --- | --- |
| Black medick | *Medicago lupulina* | Fabaceae |
| Bladder campion | *Silene vulgaris* | Caryophyllaceae |
| Bristly ox-tongue | *Helminthotheca echioides* | Asteraceae |
| Cat’s ear | *Hypochaeris radicata* | Asteraceae |
| Common mouse ear | *Cerastium fontanum* | Caryophyllaceae |
| Common vetch | *Vicia sativa* | Fabaceae |
| Creeping buttercup | *Ranunculus repens* | Ranunculaceae |
| Dandelion | *Taraxacum officinale* | Asteraceae |
| Devil’s-bit scabious | *Succisa pratensis* | Caprifoliaceae |
| Fool’s parsley | *Conium vulgare* | Apiaceae |
| Hop trefoil | *Trifolium campestre* | Fabaceae |
| Lady’s bedstraw | *Galium verum* | Rubiaceae |
| Ox-eye daisy | *Leucanthemum vulgare* | Asteraceae |
| Ribbed plantain | *Plantago lanceolata* | Plantaginaceae |
| Salad burnet | *Sanguisorba minor* | Rosaceae |
| Self-heal | *Prunella vulgaris* | Lamiaceae |
| Smooth sow thistle | *Sonchus oleraceus* | Asteraceae |
| St. John’s wort | *Hypericum perforatum* | Hypericaceae |
| Viper’s bugloss | *Echium vulgare* | Boraginaceae |
| White clover | *Trifolium repens* | Fabaceae |
| Wild basil | *Clinopodium vulgare* | Lamiaceae |

University of Brighton, Checkland Building, 4^th^ floor, south (Wildflower)

| Common name | Scientific name | Family |
| --- | --- | --- |
| Bristly ox-tongue | *Helminthotheca echioides* | Asteraceae |
| Cat’s ear | *Hypochaeris radicata* | Asteraceae |
| Common knapweed | *Centaurea nigra* | Asteraceae |
| Creeping buttercup | *Ranunculus repens* | Ranunculaceae |
| Dandelion | *Taraxacum officinale* | Asteraceae |
| Hop trefoil | *Trifolium campestre* | Fabaceae |
| Lady’s bedstraw | *Galium verum* | Rubiaceae |
| Lesser trefoil | *Trifolium dubium* | Fabaceae |
| Ox-eye daisy | *Leucanthemum vulgare* | Asteraceae |
| Petty spurge | *Euphorbia peplus* | Euphorbiaceae |
| Rosebay willowherb | *Chamerion angustifolium* | Onagraceae |
| Salad burnet | *Sanguisorba minor* | Rosaceae |
| Viper’s bugloss | *Echium vulgare* | Boraginaceae |
| White clover | *Trifolium repens* | Fabaceae |
| Wild basil | *Clinopodium vulgare* | Lamiaceae |
| Wild thyme | *Thymus serpyllum* | Lamiaceae |

University of Brighton, Checkland Building, 3^rd^ floor, south (Wildflower)

| Common name | Scientific name | Family |
| --- | --- | --- |
| Black medick | *Medicago lupulina* | Fabaceae |
| Bladder campion | *Silene vulgaris* | Caryophyllaceae |
| Bristly ox-tongue | *Helminthotheca echioides* | Asteraceae |
| Cat’s ear | *Hypochaeris radicata* | Asteraceae |
| Common corn cockle | *Agrostemma githago* | Caryophyllaceae |
| Common ragwort | *Senecio jacobaea* | Asteraceae |
| Common toadflax | *Linaria vulgaris* | Plantaginaceae |
| Creeping buttercup | *Ranunculus repens* | Ranunculaceae |
| Dandelion | *Taraxacum officinale* | Asteraceae |
| Forget-me-not | *Myosotis sp.* | Boraginaceae |
| Grass vetchling | *Lathyrus nissolia* | Fabaceae |
| Lady’s bedstraw | *Galium verum* | Rubiaceae |
| Lesser trefoil | *Trifolium dubium* | Fabaceae |
| Ox-eye daisy | *Leucanthemum vulgare* | Asteraceae |
| Petty spurge | *Euphorbia peplus* | Euphorbiaceae |
| Salad burnet | *Sanguisorba minor* | Rosaceae |
| Smooth sow thistle | *Sonchus oleraceus* | Asteraceae |
| Viper’s bugloss | *Echium vulgare* | Boraginaceae |
| White clover | *Trifolium repens* | Fabaceae |
| Wild basil | *Clinopodium vulgare* | Lamiaceae |

University of Brighton, Checkland Building, 2^nd^ floor, south (Wildflower)

| Common name | Scientific name | Family |
| --- | --- | --- |
| Bladder campion | *Silene vulgaris* | Caryophyllaceae |
| Bristly ox-tongue | *Helminthotheca echioides* | Asteraceae |
| Cat’s ear | *Hypochaeris radicata* | Asteraceae |
| Common corn cockle | *Agrostemma githago* | Caryophyllaceae |
| Common ragwort | *Senecio jacobaea* | Asteraceae |
| Common vetch | *Vicia sativa* | Fabaceae |
| Crane’s bill | *Geranium sp.* | Geraniaceae |
| Creeping buttercup | *Ranunculus repens* | Ranunculaceae |
| Dandelion | *Taraxacum officinale* | Asteraceae |
| Fool’s parsley | *Aethusa cynapium* | Apiaceae |
| Grass vetchling | *Lathyrus nissolia* | Fabaceae |
| Groundsel | *Senecio vulgaris* | Asteraceae |
| Hop trefoil | *Trifolium campestre* | Fabaceae |
| Lady’s bedstraw | *Galium verum* | Rubiaceae |
| Lesser trefoil | *Trifolium dubium* | Fabaceae |
| Lucerne | *Medicago sativa* | Fabaceae |
| Many seeded goosefoot | *Lipandra polysperma* | Amaranthaceae |
| Ox-eye daisy | *Leucanthemum vulgare* | Asteraceae |
| Petty spurge | *Euphorbia peplus* | Euphorbiaceae |
| Purple clover | *Trifolium pratense* | Fabaceae |
| Smooth sow thistle | *Sonchus oleraceus* | Asteraceae |
| Speedwell | *Veronica sp.* | Plantaginaceae |
| White clover | *Trifolium repens* | Fabaceae |
| Wild basil | *Clinopodium vulgare* | Lamiaceae |

Organic Roofs Ltd, shipping container (Wildflower)

| Common name | Scientific name | Family |
| --- | --- | --- |
| Bristly ox-tongue | *Helminthotheca echioides* | Asteraceae |
| Broad leaved dock | *Rumex obtusifolius* | Polygonaceae |
| Cat’s ear | *Hypochaeris radicata* | Asteraceae |
| Caucasian stonecrop | *Sedum spurium* | Crassulaceae |
| Dandelion | *Taraxacum officinale* | Asteraceae |
| Hoary plantain | *Plantago media* | Plantaginaceae |
| Mugwort | *Artemisia vulgaris* | Asteraceae |
| Ox-eye daisy | *Leucanthemum vulgare* | Asteraceae |
| Rosemary | *Rosmarinus officinalis* | Lamiaceae |
| White clover | *Trifolium repens* | Fabaceae |

Brighton Housing Trust, Richardson’s Yard (Wildflower)

| Common name | Scientific name | Family |
| --- | --- | --- |
| Ash | *Fraxinus excelsior* | Oleaceae |
| Crane’s bill | *Geranium sp.* | Geraniaceae |
| Hoary plantain | *Plantago media* | Plantaginaceae |
| Lady’s bedstraw | *Galium verum* | Rubiaceae |
| Ox-eye daisy | *Leucanthemum vulgare* | Asteraceae |
| Ribbed plantain | *Plantago lanceolata* | Plantaginaceae |
| Rosebay willowherb | *Chamerion angustifolium* | Onagraceae |
