# Supplementary material for: Up on the roof and down in the dirt: Differences in substrate properties (SOM, potassium, phosphorus and pH) and their relationships to each other between sedum and wildflower green roofs
Source: PLoS One. 2019 Dec 13;14(12):e0225652. doi: 10.1371/journal.pone.0225652 (PMC6910666; doi:10.1371/journal.pone.0225652)
Supplement: Attachment — Submitted filename: Response to Reviewers.docx [file pone.0225652.s003.docx]

s

Renée McAlister

c/o Dr Anja S. Rott

University of Brighton

School of Pharmacy and Biomolecular Sciences

Moulsecoomb, Brighton, BN2 4GJ

28th July 2019

Associate Professor Luitgard Schwendenmann

Academic Editor – PLOS ONE

Dear Professor Schwendenmann

I am very thankful for your kind comments on the manuscript following the submission for publication.

The manuscript has been revised in line with your comments (formatting and lines 112, 171 and 747), and I am happy to respond to your remarks below:

**7 roofs were selected and a varying number of samples (depending on the size of the roof) were sampled. What is the reasoning to consider each sample taken at a given roof as independent (n=50). In my opinion these are pseudo-replicates.**

A total of 12 roofs were surveyed, as the Checkland building had 6 individual green roofs of different heights and aspects. I was guided by the methodology of green roof researchers *Gabrych M., Kohtze, D.J & Lehvävirta, 2016. Substrate depth and roof age affect plant abundance on sedum-moss and meadow green roofs in Helsinki, Finland.* They published their work using 475 sample plots over 51 roofs, basing the sample size per roof on roof size. Ecological *in situ* studies are naturally imposed with limitations but our results indicate there was soil property variability not just between roofs, but within roofs. This was a site-specific snap shot of the soil properties of 12 green roofs in Brighton and Hove and, although its pseudo-replication has been questioned, I believe it has scientific merit as this is one of the first studies to analyse these particular four properties in green roof soils.

**Further, did you test for an age effect (time since establishment) and even more critical is the difference between the substrates. It remains unclear whether your questions relate to substrate (e.g. line 163) or plant type (e.g. 172/173).**

The ages of the roofs were recorded but not included in the statistical analysis. As this was a snap shot of the present situation on the green roofs, we did not incorporate this data. The different types of substrates were not analysed independently, only the soil samples (lines 95 – 98).). The question in regard to substrate/plant type has been addressed in line 171 which, we believe, clarifies your enquiry. The questions relate to the difference in soil properties between the two green roof types, sedum and wildflower

**Another aspect which needs to be addressed before I consider sending the manuscript for review is the difference between the carbon content at the time of establishment (baseline - carbon embodied in the substrate) and time of sampling. In my opinion it is more relevant to consider the difference (not the total amount).**

I was not able to measure the carbon baseline of the roofs at installation and nor were the owners of the roofs able to provide us with this information. Therefore this study was a scientific depiction of the present state of the roofs, rather than a comparison between their soil properties at installation and at the time of surveying.

Once more, thank you for your comments and the opportunity to respond. I look forward to hearing from you.

Sincerely

Renée McAlister
