# Supplementary material for: Up on the roof and down in the dirt: Differences in substrate properties (SOM, potassium, phosphorus and pH) and their relationships to each other between sedum and wildflower green roofs
Source: PLoS One. 2019 Dec 13;14(12):e0225652. doi: 10.1371/journal.pone.0225652 (PMC6910666; doi:10.1371/journal.pone.0225652)
Supplement: Attachment — Submitted filename: Response to Reviewers v1.docx [file pone.0225652.s004.docx]

s

Renée McAlister

c/o Dr Anja S. Rott

University of Brighton

School of Pharmacy and Biomolecular Sciences

Moulsecoomb, Brighton, BN2 4GJ

7 November 2019

Associate Professor Luitgard Schwendenmann

Academic Editor – PLOS ONE

Dear Professor Schwendenmann

I am very thankful for the kind comments of your reviewers on the manuscript following my initial revisions.

The manuscript has been revised in line with their comments. All references to soil have been changed to substrate, the missing commas (lines 52-53) have been added and the first two components of the PCA (lines 209-216) have been explained. Additionally, the wording has been changed on lines 320 and 400 in accordance with their suggestions (line 320 caveat has been changed to submit and line 404 connoting has been changed to suggesting).

**However, I don’t know how any conclusions can be made that sedum may be better than wildflowers at sequestering carbon. Are the differences in all the variables due to the plant type or to the differences in the soil components?**

I agree with the reviewers in regard to my conclusions about carbon sequestration as these conclusions were rather over speculative. I have therefore removed all references to this inference.

**Also, there is little information on plants. What is a sedum roof and what is a wildflower roof? Any grasses? Plant species could have a major impact on soil properties. A list of plant species should be included to describe each roof.**

I have included lists of all plant species on all roofs in Supporting Information 2 (signposted in Table 1). Additionally, I have added an explanatory sentence in the introduction (lines 97-100) giving a brief overview of the differences between sedum and wildflower green roofs.

**The soils on the sedum roofs were composed of recycled bricks while the soils on the wildflower roofs were composed of an assortment of different aggregates and composts. Differences in SOM, pH, and P likely existed before plants were even installed. Maybe plant type had no effect.**

I believe I have addressed this concern in lines 256-260 by qualifying that the research is presented for consideration as a snapshot of the substrate characteristics at the time of study. I have also added information that comparing the initial substrates in regard to SOM would be beneficial (lines 270-272) and included information regarding opposing studies which report either increasing or decreasing levels of SOM over time on green roofs (lines 273-276).

Additionally, Thuring & Dunnett (2014) suggested that green roof substrates do change over time in regard to their characteristics and therefore I believe this snapshot is a good representation of the current substrate characteristics taking aging into consideration. The roofs I studied were between 2 and 7 years of age, and Thuring & Dunnett (2014) indicate that substrate characteristics can indeed alter in a 2 year time frame.

Once again, thank you very much for your comments and the opportunity to respond. I look forward to hearing from you.

Sincerely

Renée McAlister

Thuring, C.E. & Dunnett, N., 2014. Vegetation composition of old extensive green roofs (from 1980s Germany). *Ecological Processes.* 3 (4) pp.1-11.
